# Supplementary material for: Involvement of Epithelial–Mesenchymal Transition Genes in Small Cell Lung Cancer Phenotypic Plasticity
Source: Cancers (Basel). 2023 Feb 25;15(5):1477. doi: 10.3390/cancers15051477 (PMC10001072; doi:10.3390/cancers15051477)
Supplement: Supplementary file 1 [file cancers-15-01477-s001.zip › MS_EMT_SCLC_SupplementaryFigures.pdf]

Supplementary Figures

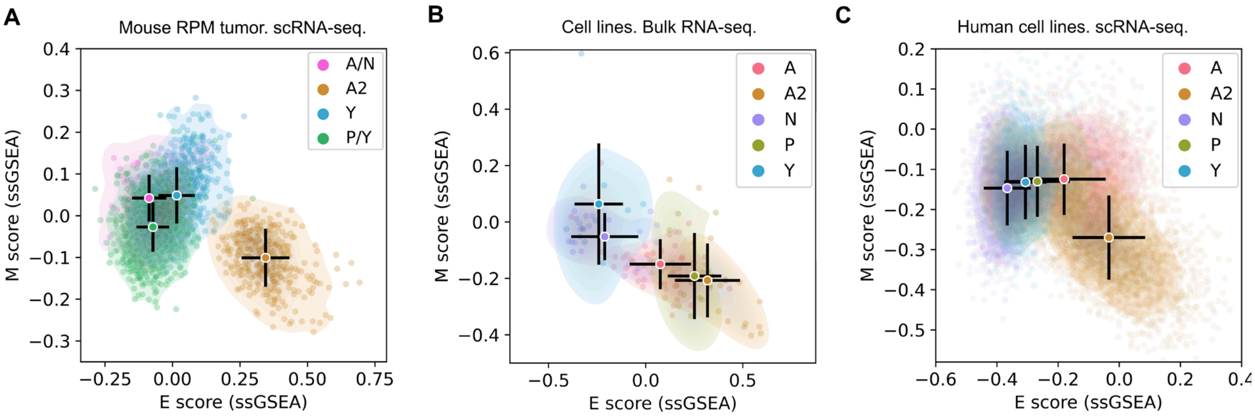

Figure S1. **Distributions of SCLC cells across the epithelial and mesenchymal spectrum using non-overlapping gene sets**. **A**. E and M scores of 15138 mouse *Myc*-driven tumor cells in an *ex vivo* model. Archetype analysis was performed to determine generalist cells and specialist cells of four types (SCLC subtypes). Scores were computed with ssGSEA and 405 previously identified EMT genes that were not used for SCLC subtyping. **B**. E and M scores of 120 SCLC cell lines computed with the same method as in A. **C**. E and M scores of 13945 cells from 8 SCLC cell lines computed with the same method as in A.

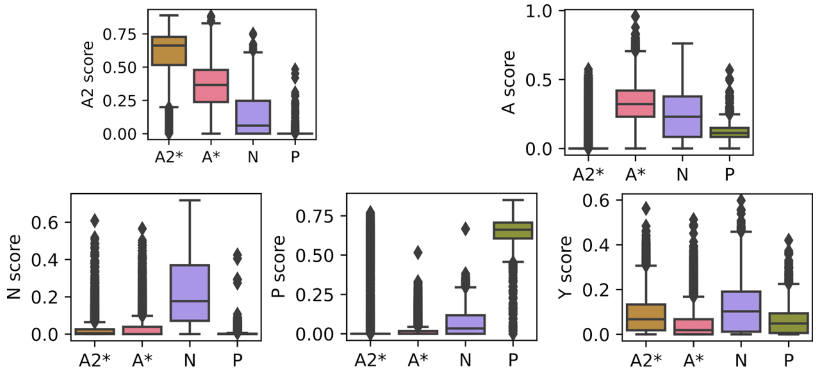

Figure S2. Subtype scores for human tumor cells. Boxplots show subtype scores including newly defined A* and A2* subtypes based on the threshold indicated in Figure 3C and D, as well as N and P subtypes defined by Chan et al. [1].

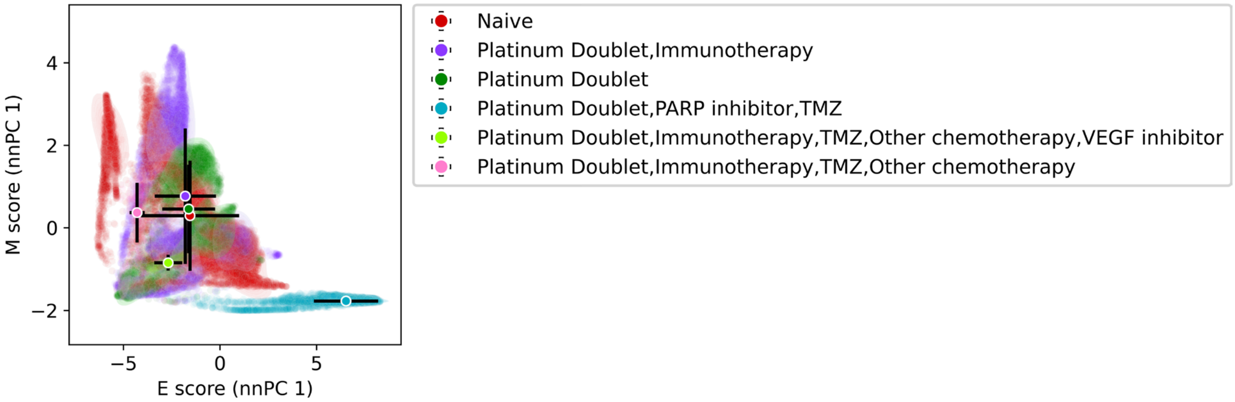

Figure S3. Treatments of human tumor cells visualized in EMT space. Scatter plot shows nnPCA-based E and M scores for 54,523 SCLC cells (Chan *et al.*, 2021). Color code represents each of the treatment types. Circles and black bars show means and standard deviations of cell scores for each treatment type.

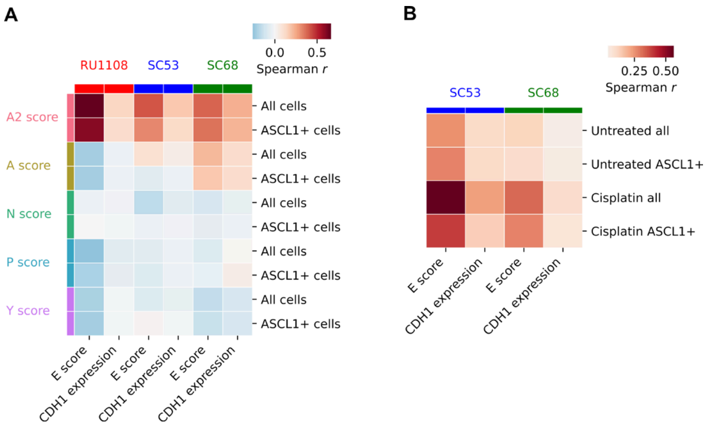

Figure S4. Correlations between A2 and epithelial transcriptional programs in individual human tumor cells using non-overlapping gene sets. **A**. Datasets of three tumors (top labels) were used to compute the Spearman correlation coefficients between SCLC subtype scores (left labels) and E scores from nnPCA or *CDH1* expression levels. All cells and ASCL1^{+} cells were analyzed separately. **B**. Datasets of SC53 and SC68 tumors were used to compute the Spearman correlation coefficients between SCLC subtype scores (left labels) and E scores from nnPCA or *CDH1* expression levels. All cells and ASCL1^{+} cells were analyzed separately. Untreated and cisplatin-treated cells were analyzed separately.

References

1. Chan JM, Quintanal-Villalonga Á, Gao VR, et al. Signatures of plasticity, metastasis, and immunosuppression in an atlas of human small cell lung cancer. *Cancer Cell* 2021; **39**(11): 1479-96.
